# Selection of high nitrogen fixation chickpea genotypes under drought stress conditions using multi-environment analysis

**DOI:** 10.3389/fpls.2025.1490080

**Published:** 2025-04-07

**Authors:** Tawffiq Istanbuli, Alsamman M. Alsamman, Khaled Al-Shamaa, Ahmed Abu Assar, Muhammed Adlan, Tapan Kumar, Sawsan Tawkaz, Aladdin Hamwieh

**Affiliations:** ^1^ Department of Biotechnology, International Center for Agricultural Research in the Dry Areas (ICARDA), Beirut, Lebanon; ^2^ Genome Mapping, Agricultural Genetic Engineering Research Institute (AGERI), Agricultural Research Center (ARC), Giza, Egypt; ^3^ Department of Genetic Innovation, International Center for Agricultural Research in the Dry Areas (ICARDA), Beirut, Lebanon; ^4^ Department of Oil Crops, Agriculture Research Center (ARC), Wad Madani, Sudan; ^5^ Department of Chickpea Plant Breeding, International Center for Agricultural Research in the Dry Areas (ICARDA), Amlaha, India

**Keywords:** nitrogen fixation, drought stress, multi-environment analysis, chickpea, selection lines

## Abstract

**Introduction:**

Chickpea (*Cicer arietinum* L.) is an important pulse crop mainly grown in marginal lands around the world. Drought stress highly impacts symbiotic nitrogen fixation (SNF) in chickpeas, which can limit productivity. Therefore, selecting high nitrogen fixation chickpea genotypes that can tolerate water stress is important for breeding programs.

**Methods:**

A total of 204 chickpea genotypes were assessed in eight different environments across Lebanon during the 2016 and 2017 growing seasons, under both rainfed and irrigated conditions. The study employed an Alpha Lattice design with two replications at two distinct locations. Data were collected for yield and nodule characteristics, then subjected to AMMI and GGE biplot analysis.

**Results and Discussion:**

The AMMI analysis indicated that genotype (G), environments (E), and genotype × environment interaction (GEI) had significant effects on grain yield (P<0.001), highlighting the presence of genetic variation and the potential for selecting stable genotypes. The findings revealed that the environmental effect predominantly influenced chickpea grain yield, with GEI following, and G having the least impact. Environment explained 34.5% of the total (G + E + GE) variation, whereas G and GEI captured 16.4% and 24.3%, respectively. According to grain yield (GY), genotype IG70399 demonstrated the highest performance across all environments, while genotype IG8256 displayed the most consistent performance across different conditions. In a rainfed environment, genotype IG73394 had higher nodulation, while IG70384 and IG70410 had higher nodulation biomass (NB) under an irrigated environment. The NB for ten highly tolerant genotypes increased by 24% compared to the two susceptible genotypes under drought stress conditions, while the NB for these ten genotypes increased by 14.6% compared to all studied genotypes.

## 1 Introduction

Chickpea (*Cicer arietinum L.*) stands as one of the most significant grain legumes cultivated and consumed globally, particularly in tropical and subtropical regions of Afro-Asian countries. It is ranked third among food legumes in terms of world production (Merga and Haji, 2019), with an average yield of 965 kg ha-1 and a total production of 17.2 million tons was produced on 17.8 million hectares in 2019 (FAO, 2020). Currently, chickpea is grown in over 50 countries across the Indian subcontinent, North Africa, the Middle East, southern Europe, America and Australia (Nasr Esfahani et al., 2014). This legume has been considered as a beneficial source of proteins, carbohydrates, minerals, vitamins and health-promoting fatty acids (Begum et al., 2023). Its seeds, composed of approximately 21% protein, are a major source of protein for millions of families in developing countries (Merga and Haji, 2019).

As a leguminous crop, chickpea possess a significant ability to fix atmospheric nitrogen (N_2_) through a symbiotic relationship with compatible Mesorhizobium soil bacteria, the common chickpea-specific rhizobia species. Symbiotic N2 fixation is the major route for providing a large nitrogen proportion for human consumption and animal feed and contributes to agriculture sustainability (Nasr Esfahani et al., 2014; Bhattacharyya and Jha, 2012). Thus, chickpea can obtain over 70% of its nitrogen requirement through symbiotic nitrogen fixation (SNF) Flowers et al. (2010), by fixing up to 140 kg atmospheric N2 ha-1 (Nasr Esfahani et al., 2014). Although the average of chickpea yield production in the world is 965 kg ha-1 (FAO, 2020) and The productivity has consistently risen since 1961; however, its susceptibility to biotic and abiotic stresses has also heightened, likely due to the repeated use of a limited number of germplasm accessions and donor parents (Merga and Haji, 2019). Among the environmental stresses affecting productivity, Drought is one of the most important constraints limiting yield potential in cereal (Samarah, 2005; Istanbuli et al., 2020) and legume crops (Leport et al., 2006; Maqbool et al., 2017). It can reduce chickpea yields by up to 70% (Molina et al., 2008; Varshney et al., 2014, 2019). A major challenge for crop breeders is increasing yields to feed the estimated 10 billion people globally by 2050. Therefore, the development of drought-tolerant chickpea varieties has been considered as one of the major aims of chickpea breeding programs, which require an in-depth understanding of the physiological and biochemical mechanisms involved in the regulation of chickpea response to drought (Molina et al., 2008).

Studying the stability and preferred responses of genotypes across various environments is essential for plant breeders (Yang et al., 2009; Maqbool et al., 2017). Genotype x Environment Interaction (GEI) is a critical factor for plant breeders and agronomists aiming to forecast cultivar performance across various locations and years. GEI may occur when specific genotypes are cultivated in a range of environments. A notable G × E interaction regarding quantitative traits, such as seed yield, can significantly hinder the selection of superior genotypes for both new crop production and cultivar development (Kang and Gorman, 1989). Typically, environmental factors account for the majority of total yield variation, while the influence of genotype and Genotype × Environment Interaction (GEI) is comparatively minimal (Yan and Kang, 2003; Dehghani et al., 2008). Various methodologies have been proposed to evaluate the stability of genotypes across different environments. Biplot was suggested as an acceptable methodology for evaluating genotypes in various target contexts (Yan et al., 2007). Two forms of biplots, the AMMI biplot (Gauch, 1988) and the GGE biplot (Yan et al., 2000, 2007), are commonly used to illustrate genotype x environment interaction. The multivariate model, AMMI, appeared to be capable of extracting a significant portion of the genotype x environment interaction and was effective in assessing interaction patterns (Zobel et al., 1988). Breeders can now evaluate all aspects of the data more thoroughly and visually with the more recent method, the GGE (genotype main effect (G) plus G x E interaction) biplot model. This method creates a biplot that simultaneously represents mean performance and stability, as well as identifying mega-environments (Yan and Holland, 2010; Yan et al., 2000). As a result, the breeding program multi-environments data structure may be effectively analyzed and commented upon using the AMMI and GGE biplot models (Yan et al., 2000; Zobel et al., 1988).

This study aims to assess the performance of 204 genotypes of chickpeas under various environmental situations, such as rainfed and irrigated environments, to assess the impact of drought stress on symbiotic nitrogen fixation (SNF) in chickpea, and to identify high nitrogen fixation chickpea genotypes that can tolerate water stress for use in breeding programs. The purpose of the study is to discover stable genotypes that can function effectively in a variety of contexts as well as to ascertain the effects of genotype (G), environment (E), and genotype x environment interaction (GEI) on grain production. Along with evaluating the nodule properties of the various genotypes, the study seeks to find genotypes that exhibit high nodulation biomass (NB) and stability in drought-stressed environments.

## 2 Materials and methods

### 2.1 Experimental design

**Figure 1:**
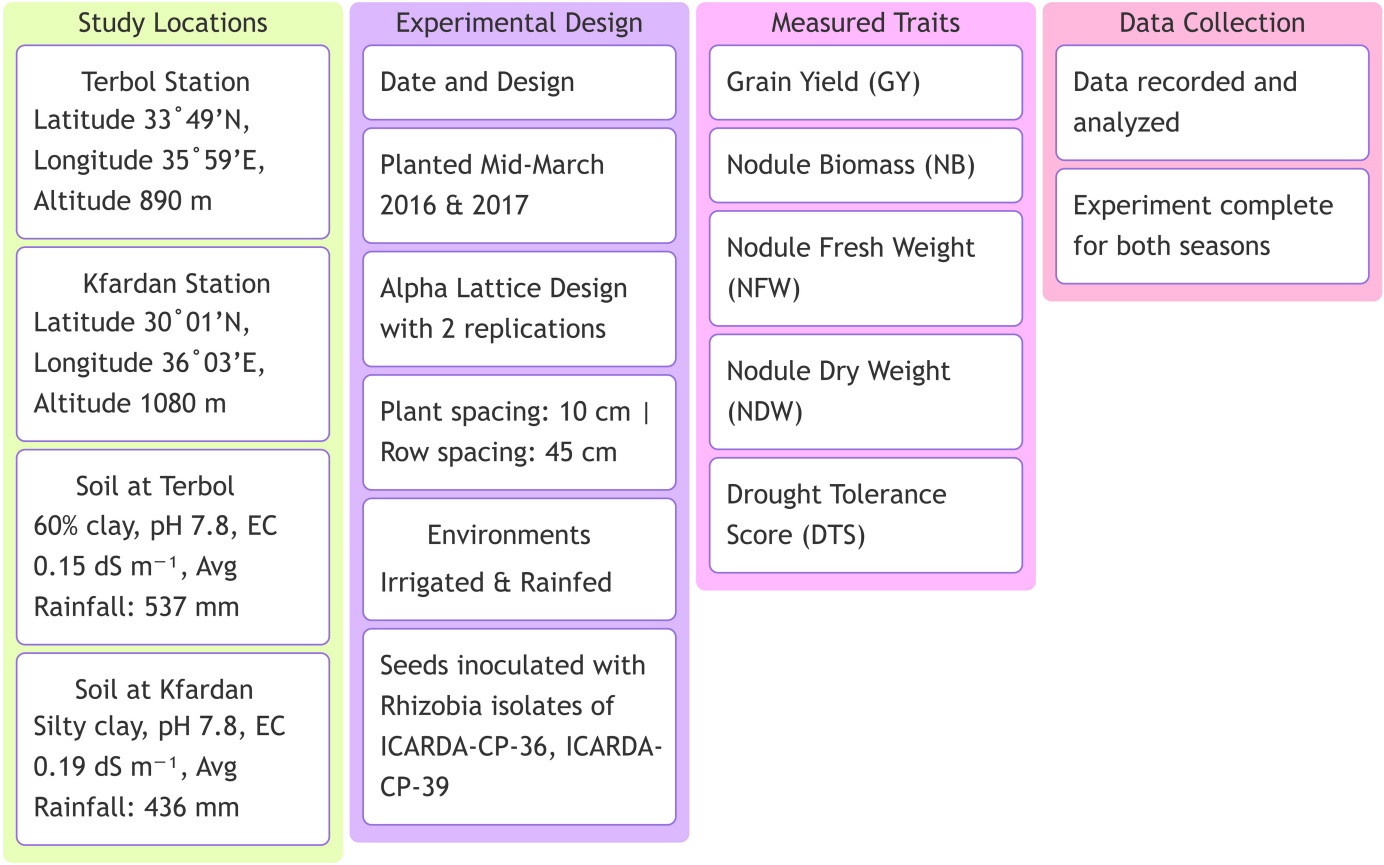
Experimental setup and key measurements for the study on high nitrogen fixation chickpea genotypes under drought stress.

This study was carried out at the International Center for Dry Area Agricultural Research (ICARDA), the Terbol station (Latitude 33° 49’ N and Longitude 35° 59’ E at an altitude of 890 m above the mean sea level) and the Kfardan station (Latitude 30° 01’ N and Longitude 36° 03’ E at an altitude of 1080 m above the mean sea level) (**Figure 1**). Based on passport data, the focused germplasm identification strategy (FIGS) was used to identify 204 genotypes from the Genetic Resource Section (GRS) of ICARDA. **Supplementary Table Sa** displays information about the 204 genotypes. The crop was sown in the middle of March in 2016 and 2017 season using Alpha Lattice design with two replications in both irrigated and rainfed environments (**Table 1**). The distance from plant to plant was 10 cm with a total of 25 plants/row and the distance from row to row distance was 45cm. The average rainfall was about 537 mm, 436 mm in Terbol and Kfardan stations, respectively. Terbol soil is 60% clay, with a pH of approximately 7.8 and a EC of approximately 0.15 dS m^−1^. Kfardan soil is silty clay, with a pH of approximately 7.8 and a CE of approximately 0.19 dS m^−1^. During the crop growing seasons (second week of March, April, and May), total rains were approximately 106.6 mm compared to 61.8 mm during 2017, while total rains in the Kfardan location were 43.2 and 59.6 mm during the 2016 and 2017 seasons, respectively. Seeds were inoculated with two kinds of Rhizobia (ICARDA-CP-36 and ICARDA-CP-39). The purpose of inoculating all genotypes in the field was to ensure homogeneity in the treatment of all genotypes of chickpeas. At harvest time seed yield and nodule characteristics were determined for each genotype at each test environment. Data were recorded for grain yield (GY) and nodule characteristics such as nodule biomass (NB), fresh nodule weight (NFW), dry nodule weight (NDW) and drought tolerance score (DTS). A total of three plants per entry were selected for data recording to these parameters.

**Table 1:** The field experiment for drought tolerance was conducted in two locations and two water treatments in the cropping seasons of 2016 and 2017.

Environments	Year	Location	Water treatment	Rainfall (in mm)
1	2016	Terbol	Rainfed	106.6
2	2016	Kfardan	Rainfed	43.2
3	2017	Terbol	Rainfed	61.8
4	2017	Kfardan	Rainfed	59.6
5	2016	Terbol	Irrigated	106.6
6	2016	Kfardan	Irrigated	43.2
7	2017	Terbol	Irrigated	61.8
8	2017	Kfardan	Irrigated	59.6

### 2.2 Statistical analysis

GenStat 19th edition statistical software was used for the statistical analysis (GenStat for Windows, 2017) (**Figure 1**). The grain yield and nodule characteristics data were subjected to a combined analysis of variance across 8 environments (2 locations: Terbol and Kfardan, 2 years: 2016 and 2017, and 2 water stress treatments: rainfed and irrigated). The AMMI (additive main effects and multiplicative interaction) model (Zobel et al., 1988) was used in a combined analysis of variance to divide the total variation into components owing to the environment (E), interaction effects (G x E), and genotype (G). In order to determine superior genotypes and evaluate GY and NB stability, chickpea genotypes, environments, and interactions were evaluated using GGE biplot analysis (Yan et al., 2000; Yan and Kang, 2003). Scatter biplot, ‘which won where’ biplot, comparison biplot for environments and genotypes were drawn for GY and NB traits to study the G x E interaction among genotypes and environments. The genotypes were classified into representative groups based on the mean grain yield, nodules characteristics, and drought tolerance score by hierarchical cluster analysis using Ward’s method (Murtagh and Legendre, 2014). The drought tolerance scores (DTS) were designed by ICARDA (Sabaghpour et al., 2006) for the assessment of drought tolerance in chickpea as a score (1–9) at the maturity stage. 1 = free, 2 = highly tolerant, 3 = tolerant, 4 = moderately tolerant, 5 = Intermediate tolerant, 6 = moderately susceptible, 7 = susceptible, 8 = highly susceptible, 9 = 100% death.

## 3 Results

### 3.1 Drought response categorization

**Figure 2:**
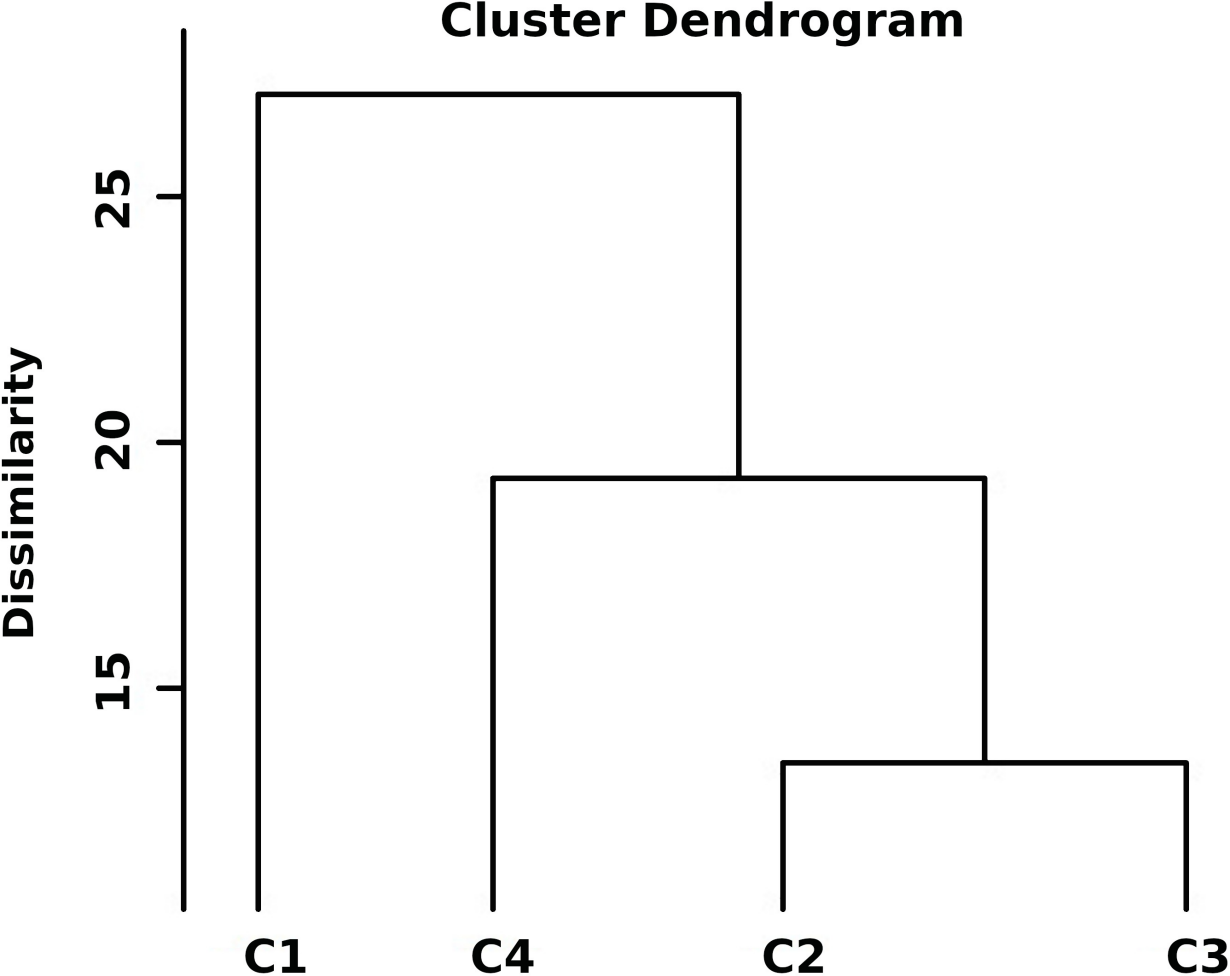
Hierarchical clustering of standardized drought traits for 204 chickpea genotypes using Euclidean distance and Ward’s method. Class1 (C1) = highly drought tolerance, class2 (C2) = drought sensitive, class3 (C3) = moderate tolerant, and class4 (C4) = tolerant.

The hierarchical cluster analysis of 204 diverse chickpea genotypes based on standardized DTS, GY, NB, NDW and NFW was done using Euclidean distance and Ward’s method (**Supplementary Figures S1**), the dendrogram was cut such that exactly four representative clusters were produced (**Figure 2**). Based on the amount of cluster group means of drought features, the following can be identified: (DRhT) highly tolerant (with means 4.13, 14.40, 4.36, 0.68 and 4.49 for DTS, GY, NB, NDW and NFW, respectively), (DRS) drought sensitive (6.46, 5.53, 0.91, 0.16 and 0.91), (DRmT) moderately tolerant (5.29, 8.72, 1.34, 0.23 and 1.38), (DRT) tolerant (5.28, 8.52, 2.25, 0.38 and 2.32) (**Supplementary Table S1**).

**Figure 3:**
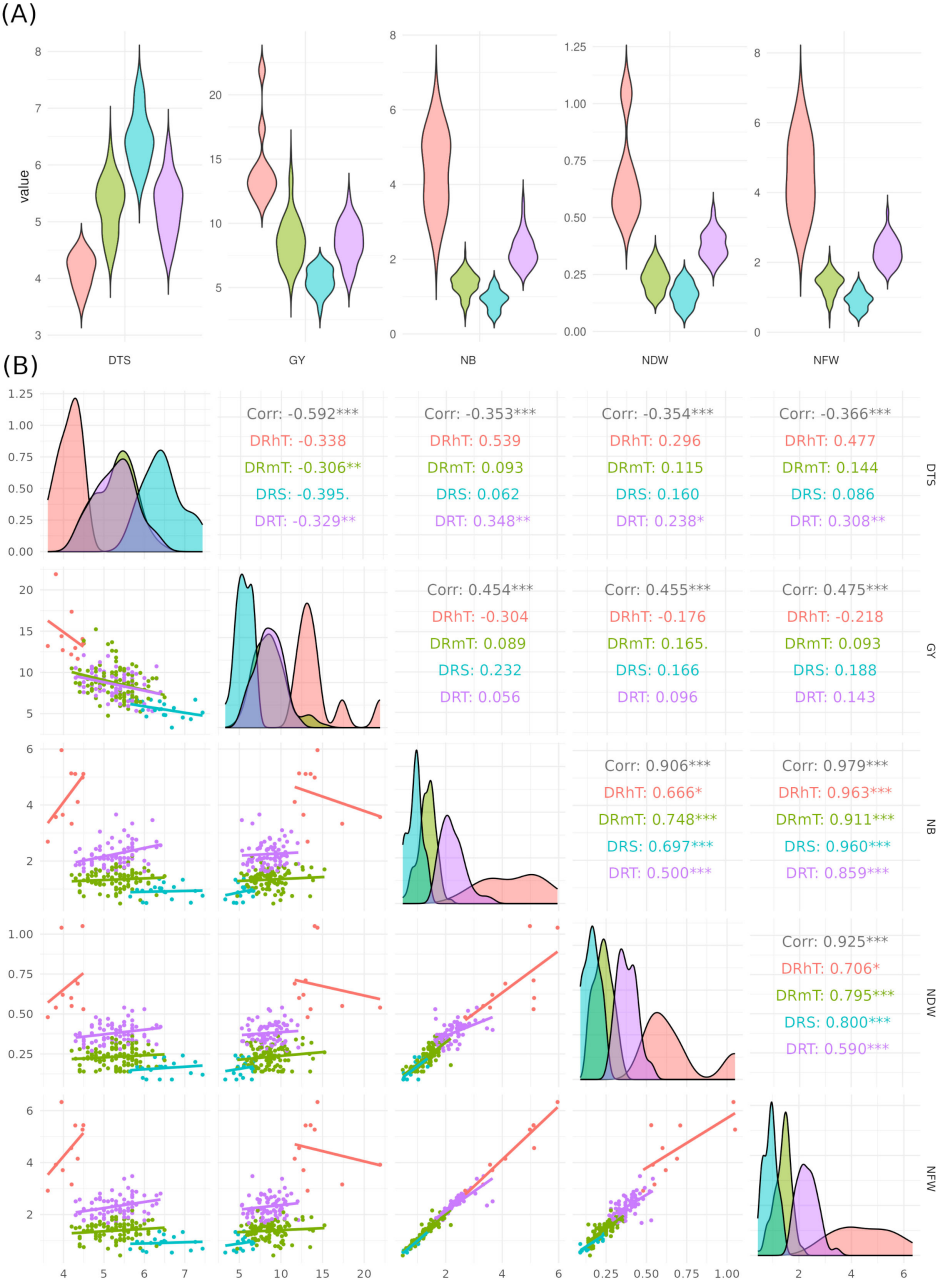
**(A)** Violin box-plots showing the distribution of key phenotypic traits across chickpea genotypes under four drought tolerance levels: highly tolerant (DRhT), sensitive (DRS), moderately tolerant (DRmT), and tolerant (DRT). **(B)** Correlation matrix with density plots and scatter plots, illustrating trait relationships. Colored correlation values indicate different treatments: red/orange for highly tolerant (DRhT) and moderately tolerant (DRmT) conditions and green/blue for sensitive (DRS) and tolerant (DRT) genotypes. Scatter plot points are colored by genotype groups or stress levels (*P *<* 0.05; **P *<* 0.01; ***P *<* 0.001).

There were 74 genotypes in the tolerant group and 101 genotypes in the somewhat tolerant group (**Supplementary Table S1**). The NB for ten highly tolerant genotypes increased by 24.4% compared to the two susceptible genotypes under drought stress conditions, while the NB for these ten genotypes increased by 14.6% compared to all studied genotypes. The distribution of key phenotypic traits across chickpea genotypes under different conditions (**Figure 3A**). Notably, NB distributions highlight certain genotypes with higher values under irrigated conditions (**Figure 3A**). The GY distribution further emphasizes the dominant influence of environmental factors on yield performance. The distinct trait distributions suggest that specific genotypes exhibit superior performance under stress, which underscores the potential for selecting highnitrogen-fixing, drought-tolerant chickpea genotypes for breeding programs. The phenotypic correlation coefficients among the quantitative traits under rainfed and irrigation conditions are presented in **Figure 3B**. DTS had a significant negative correlation with GY and nodules traits, whereas GY had a significant positive correlation under two water levels. A highly significant and positive correlation was found among nodule characteristics (**Figure 3B**).

### 3.2 Combined analysis of variance

The results of AMMI variance analysis and linear regression for the grain yield of 204 genotypes of chickpeas were tested in eight settings **Table 2**. According to **Table 2**, AMMI analysis revealed that there was a highly significant difference at level P *<* 0.001 in the variation among E, G, and G×E interaction. The SS partitioning revealed that the environment effect, GE, and genotype effect were the main sources of variation. Environments accounted for 34.5% of the total variation (G + E + GE), while genotype and the G×E interaction accounted for 16.4% and 24.3% of the variation, respectively. Additionally, **Table 2**’s AMMI analysis results showed that both IPCA1 and IPCA2 AMMI were very significant (P *<* 0.001). The GEI sum of squares was explained by the first and second principal component axes, respectively, in 38.9% and 21.4% of cases. The combined mean squares of the IPCA1 and IPCA2 accounted for 60.3% of the GEI. Compared to GEI, the environmental effect was about 1.4 times greater. The GEI sum of squares had a magnitude that was almost 1.5 times greater than genotypes.

**Table 2:** AMMI analysis of variance for seed yield of 204 chickpea genotypes tested at eight environments.

Source of variation	DF	Sum of squares	Mean square	F-value	SS% explained
Total	3263	153285	47		
Block	8	2145	268.2	12.04	
Treatments	1631	115401	70.8	3.18	
Genotypes (G)	203	25195	124.1	5.57***	16.4%
Environments (E)	7	52894	7556.3	28.18***	34.5%
Interactions (G x E)	1417	37312	26.3	1.18***	24.3%
IPCA 1	209	14508	69.4	3.12	38.9%
IPCA 2	207	7975	38.5	1.73	21.4%
Residuals	1001	14830	14.8	0.67	39.7%
Error	1605	35739	22.3		

*** refers to significant at <0.001 probability level.

IPCA 1 and 2 = first and second main additive axis, respectively.

### 3.3 Biplot analysis for grain yield

**Figure 4:**
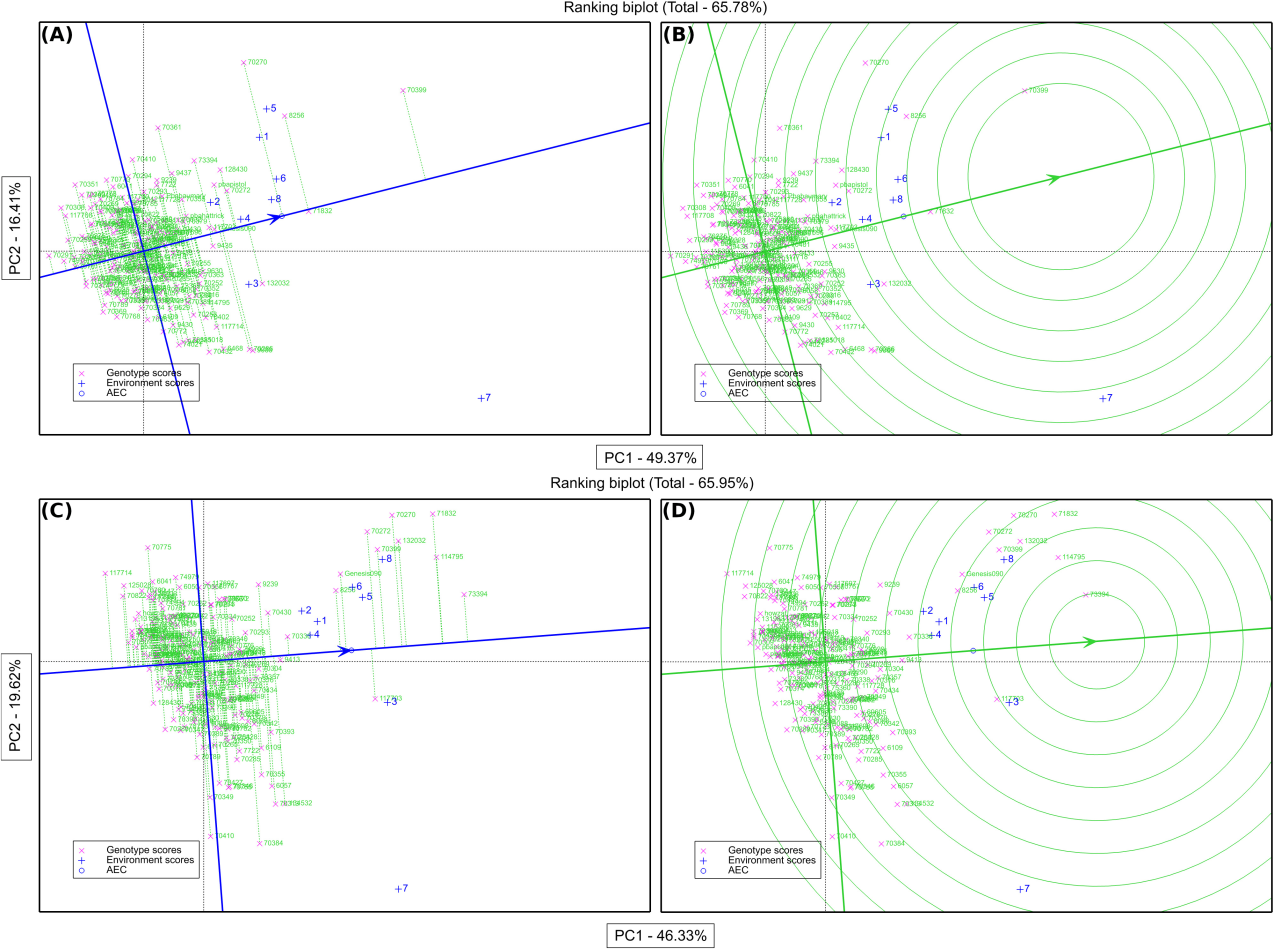
Average environment coordination (AEC) plot of the GGE-biplot based on environment-focused scaling for the means of grain yield performance, stability **(A)**, and comparison of genotypes in seed yield with the ideal genotype **(B)**. Average environment coordination (AEC) plot of the GGE-biplot based on environment-focused scaling for nodule biomass performance, stability **(C)**, and comparison of genotypes in nodule biomass with the ideal genotype **(D)**. Green and blue numbers stand for genotypes and environments, respectively.

**Figure 5:**
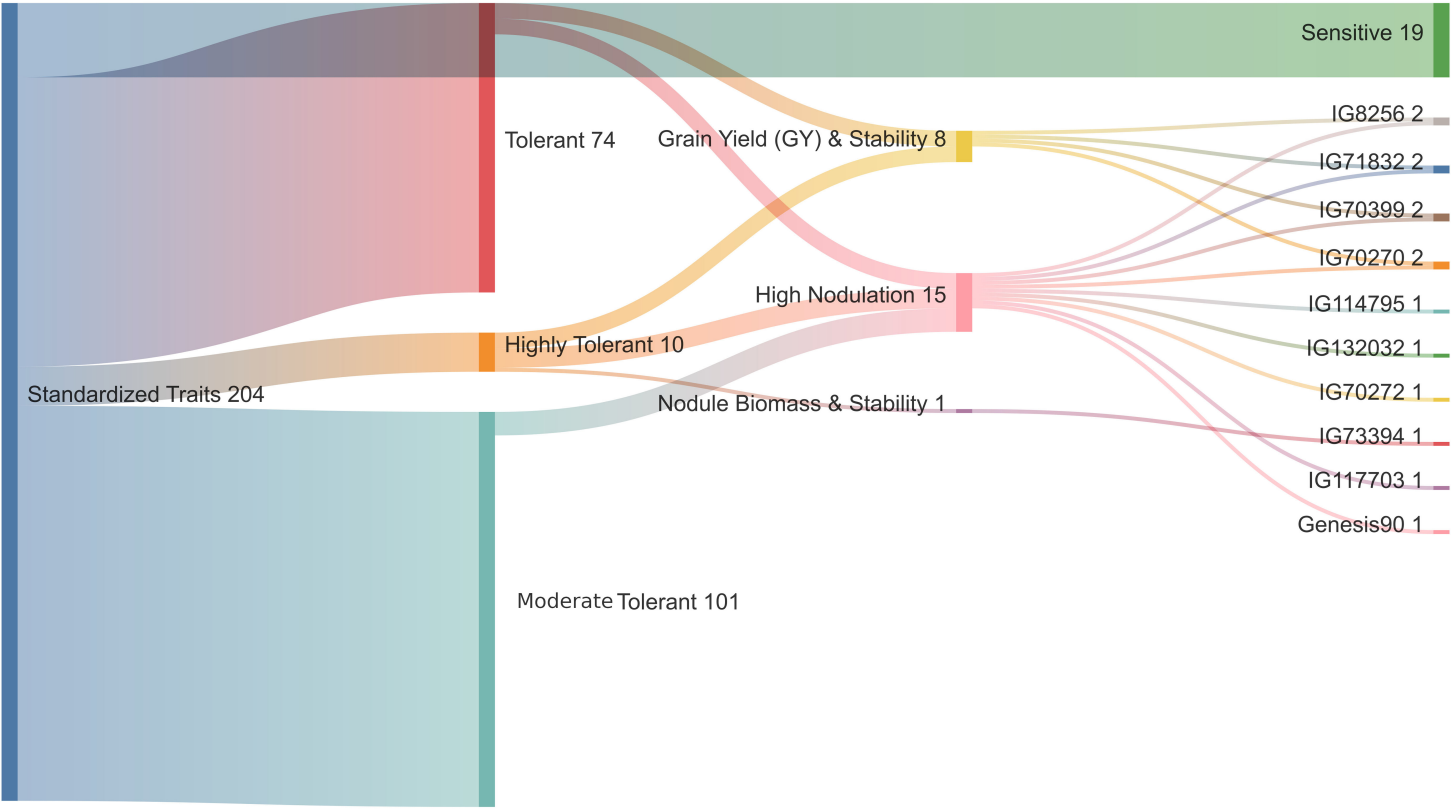
Categorization of 204 genotypes based on standardized traits, including Days to Symptom (DTS), Grain Yield (GY), Nodule Biomass (NB), Nodule Dry Weight (NDW), and Nodule Fresh Weight (NFW).

**Figure 6:**
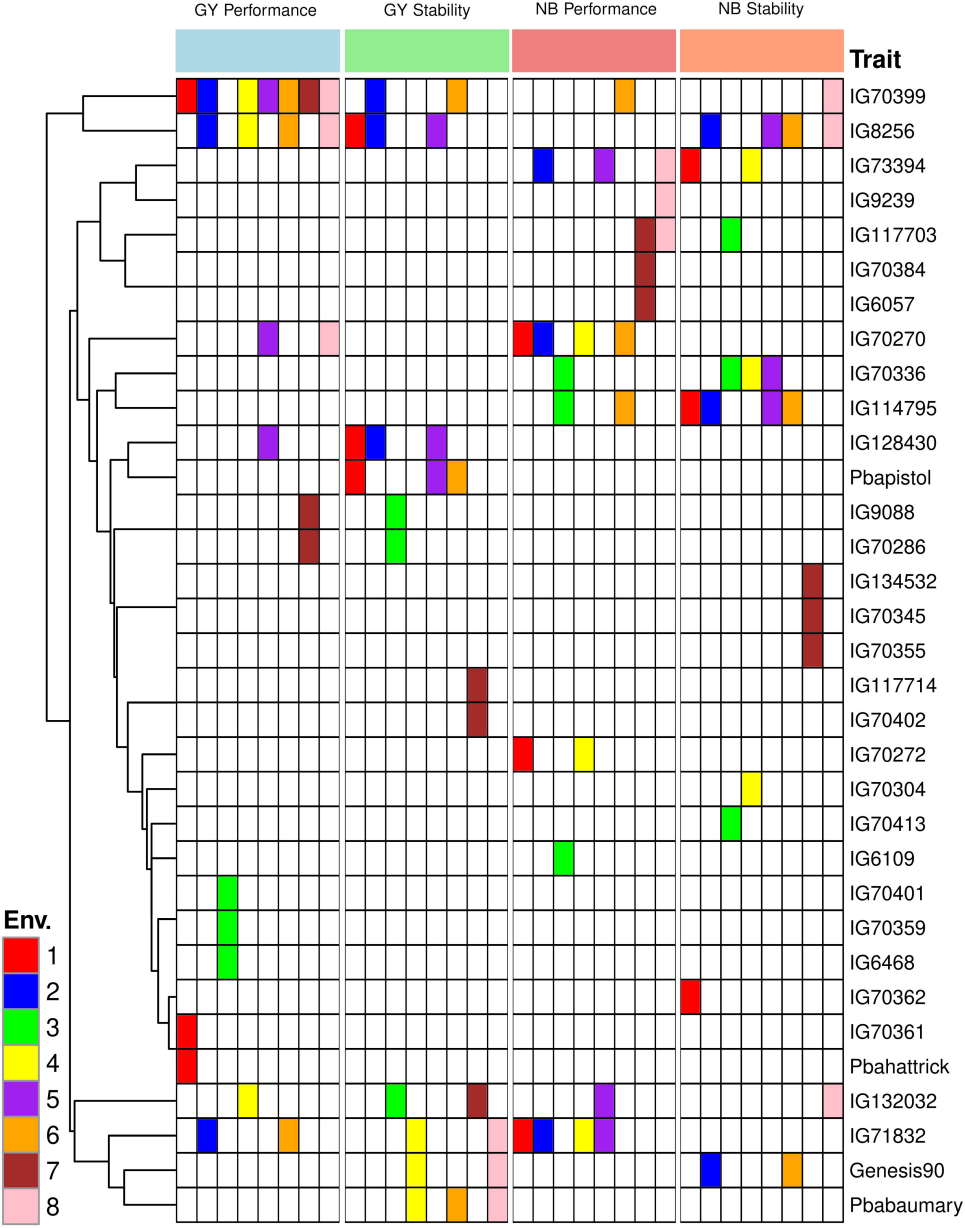
Performance and stability for grain yield and nodule biomass for the best three chickpea genotypes based on GGE biplot for studied eight environments.

The GGE biplot for grain yield accounted for 65.78% of the variation due to G main effect and G × E interaction. The primary (PC1) and secondary (PC2) components explained 49.36% and 16.42% of genotype main effects and G × E interaction respectively (**Supplementary Figure S2A**). A concise description of the links between the environments can be found in **Supplementary Figure S2B**’s vector view of the GGE-biplot. All of the environments had positive correlations because their angles were all smaller than 90° and there was correlation within each environment (1, 2, 4, 5, 6, and 8) and the environments (3 and 7) were more than between them (**Supplementary Figure S2A**). A GGE biplot’s polygon view provides a concise synopsis of the GE pattern by explicitly displaying the which-won-where pattern (**Figure 4B**). By joining the genotype markers, the rays in **Figure 4B** depict lines perpendicular to the polygon’s sides and extensions. The biplot is divided into nine sectors by nine rays, although every environment falls into one of the sectors (sector one). Given its greater distance from the biplot origin, the vertex genotype (IG70399) is the most sensitive genotype in this sector and could have a higher GY. According to our results, IG70399 was the best genotype in all environments, followed by IG8256, IG71832, and IG70270 (**Supplementary Figure S2B**). The performance and stability of grain yield was assessed using the average environment coordination (AEC) approach. Genotypes IG70399, IG71832, IG8256, and IG70270 had a higher GY and average of stability (**Figures 5**, **6**). In terms of only stability across test environments, IG8256 was the best genotype among all others. Instead of using many graphs for each GGE biplot environment, the results were presented tabulated. Nodule biomass performance and stability are presented in **Figure 4C**. The GGE biplot analysis allows comparing the test genotypes to a reference genotype. This method specifies the position of an ideal genotype with high product ability and stability. **Figure 4D** illustrates that the ideal genotype (IG70399) falls into the center of the concentric circles based on average genotype yield (**Figure 4B**).

### 3.4 Biplot analysis for nodule biomass

In addition to conducting GGE biplot analyses to assess the yield performance of these genotypes in the test environments, it was imperative to determine the N2 fixation of these genotypes in both rainfed and irrigated environments. The GGE biplot analysis for NB explained 65.95% of the genotype’s main impact and the GxE interaction. The primary (PC1) and secondary (PC2) components explained 46.33% and 19.62% of genotype main effects and G × E interaction, respectively (**Supplementary Figure S2C**) of the main impacts of genotype and the G × E interaction are shown in **Figure 4C**. In addition to assessing how well these genotypes function in the test conditions using GGE biplot analyses, it was necessary to know the N fixation for these genotypes through GGE biplot analysis for NB under rainfed and irrigated environments. Three environments (1, 3, and 4) fell into sector 1, and the vertex genotypes for this sector were IG73394, indicating a stronger nodulation cross-rainfed environment, followed by genotype IG114795. One environment (7) dropped into sector two and the vertex genotype for this sector was IG70384, which was delineated by rays 2 and 3. Four environments (2, 5, 6, and 8) fell all into sector 8 but the environment (5) has a joint point between sectors 1 and 8, and the vertex genotypes (IG71832 and IG73394) were the most responsive genotypes and may have higher NB, followed by genotypes IG114795, IG132032, IG70270, IG70399, IG70272, Genesis90 and IG8256 were also considered high nodulation genotypes under irrigated conditions (**Supplementary Figure S2C**). The average performance and stability for genotypes in nodule biomass are shown in **Figure 4C**. The results revealed that genotype IG73394 had higher nodule biomass and stability, whereas other genotypes (IG71832, IG114795, IG70270, IG132032, IG70272, IG70399, IG117703, Genesis90 and IG8256) with higher nodulation in the test environments (**Figure 6**). A superior genotype with lots of nodules and good stability is one that is found in the middle of the circles or is the genotype that is most similar to the ideal genotype. Based on its closest match to the ideal genotype, genotype IG73394 was determined to be the best genotype in nodule biomass (**Figure 4D**).

## 4 Discussion

Assessing genotypes requires a thorough understanding of the relationship between genetics and environment. Studying interactions became simpler with the development of biplot graphical analysis. In a biplot, a two-way table is shown that illustrates the relationship between column factors, row factors, and their interaction within a single platform (Gabriel, 1971; Yan, 2001). The results of the AMMI ANOVA for seed yield of 204 genotypes in the 8 settings showed substantial variations in the effects of genotypes, habitats, and interactions (p *<* 0.001; **Table 2**). Similar findings were reported by many writers (Jacobsz et al., 2015; Erdemci, 2018; Tadesse et al., 2017; Adane and Abebe, 2018), indicating the presence of significant heterogeneity among genotypes, settings, and the potential for selection for stable genotypes.

**Table 2** indicates that the effects of the environment were greater than the GxE interaction, indicating the potential presence of distinct environment groups (Erdemci, 2018). **Table 2** shows that the GEI effect is about twice as large as the genotypic effects, suggesting that genotypes respond differently to environmental changes and that settings can discriminate against one another (Farshadfar et al., 2011; Yan and Rajcan, 2002). The outcomes revealed a positive relationship within environments 1, 2, 4, 5, 6, and 8 and environments 3 and 7. However, there was less of a correlation between them, indicating that grain yield can be indirectly selected in a variety of test settings. Regarding favorable rain distribution in Terbol-2017, environments 3 and 7 differed from other settings in terms of which would positively affect the yield and nodule biomass performance in this environment (**Supplementary Figure S2A**).

A polygon view of the biplot drawn on genotypes illustrates that while most genotypes are inside the polygon, a few are positioned at the vertices (**Supplementary Figure S2B**). The vertex genotype IG70399 demonstrated the highest responsiveness, indicating that it performed either exceptionally well or poorly in specific environments due to its extreme position from the origin (Yan and Rajcan, 2002; Farshadfar et al., 2011). Among the tested genotypes, IG70399 exhibited the best overall yield performance across both rainfed and irrigated conditions, followed by IG8256 and IG71832. These vertex genotypes, particularly IG70399, are potential candidates for cultivation in the studied environments due to their superior yield potential. Since all test environments fall within the same sector, the vertex genotypes in this sector likely represent the highest-yielding performers under these environmental conditions (Yan and Rajcan, 2002). An average environment coordination (AEC) approach was used to assess the genotype yield stability (Yan, 2001; Yan et al., 2000; Yan and Rajcan, 2002). The results generated from this study revealed that genotype IG70399 had a higher average yield compared to other genotypes in the test environments. However, genotype IG71832 exhibited more stability in the studied environments (**Figure 4A**).

GGE biplot provided both aspects under the same umbrella, both mean performance and stability can be assessed in one graph, as reported by Yan and Kang (2003). For the purpose of growing chickpea, genotypes with a high seed output and generally consistent performance are crucial. Thus, it is best to choose genotypes that are broadly suited to various test conditions. The yield performance and stability of various genotypes for each environment were shown by the GGE biplot analysis (**Figure 6**). In order to assess the average performance and stability of the genotypes, AEC was plotted (Yan, 2001; Yan and Hunt, 2002). The genotype by environment interaction is described by PC2 in the GGE biplot, while PC1 reflects the genotypic average performance, which was used as a measure according to Yan et al. (2000). In this study, the genotype with the highest seed yield and stability was found to be IG70399, as depicted in **Figure 6**. The other genotypes, IG71832 and IG8256, also showed promising results and were ranked next in line. Yan and Kang (2003) state that the optimal genotype should have the best mean performance and stability; this genotype is shown in **Figure 4B** as a dot with an arrow pointing to it. Previous findings state that the optimal genotype is a hypothetical genotype which has a higher mean yield and stable yield (Yan and Rajcan, 2002). The ideal genotype has a large PC1 score (high mean yield) and very low PC2 value (high stability). For choosing genotypes that are stable and produce high yields, GGE biplot analysis optimum genotype position procedure is advised as the best approach (Maqbool et al., 2015).

In addition, to analyze the GGE biplot for GY of these genotypes in the test environments, it was necessary to know the N fixation through GGE biplot analysis for NB under rainfed and irrigated conditions. The polygon for NB trait of 204 chickpea genotypes under 08 environments is shown in **Supplementary Figure S2C**. The biplot in this study is divided into 09 sectors by 09 rays. However, the environments are located in only three of these sectors, indicating that the genotype’s vertex in these sectors may have higher or the highest nodulation across all environments (Yan and Rajcan, 2002). The vertex genotype IG73394 in the sector 1 followed by genotype IG114795 had a higher nodule biomass in rainfed environments (1, 3 and 4), while the vertex genotype IG70384 followed by genotype IG70410 in the sector 2 are considered a superior genotype in irrigated environment (7). The sector 8 had the vertex genotype IG71832 was the most responsive genotype and may have high NB, followed by genotypes IG73394, IG114795, IG132032, IG70270, IG70399, IG70272, Genesis90 and IG8256, which also had higher nodulation and suitable for rainfed and irrigated environments (**Supplementary Figure S2C**). The genotypes’ performance and stability were evaluated by an average environment coordination (AEC) method (Yan, 2001; Yan et al., 2000; Yan and Rajcan, 2002). The genotype IG73394 had the highest nodulation and stability in the test environments (**Figure 4**). The genotype that was found to be the most desirable was IG73394, which was located closer to the ideal genotypes found in the study (**Figure 4B**) According to the preliminary results of this study, the genotypes IG70399, IG73394, IG8256, IG71832, IG132032, IG70272, IG70270, IG114795, Genesis90 and IG117703 showed high performance and relatively stable in the test environments. They were selected and used for breeding programs (**Figure 6**). This study indicated that plants with higher nodule biomass and a higher number of pods per plant have higher grain yield. These traits could be used effectively for screening high yielding genotypes under drought stress conditions. Similar results were also reported by Istanbuli et al. (Istanbuli et al., 2022). To this end, emphasis should be given to developing chickpea genotypes with high growth rates and nodulations to improve grain yield.

## 5 Conclusion

The AMMI analysis’ findings indicated that while genotype had the least influence on chickpea grain production performance, environmental factors had the biggest impact, followed by GEI. The GGE biplot analysis revealed that IG8256 was the most stable genotype, whereas IG70399 had good performance and stability in grain yield. Because they were closest to the optimal genotype, genotypes IG70399 and IG73394 were found to be the best for grain yield and nodule biomass, respectively. Choosing the best possible genotypes is crucial in breeding programs as they possess high yield and nodule values with stable performance. The genotypes IG70399, IG73394, IG8256, IG71832, IG132032, IG70272, IG70270, IG114795, Genesis90, and IG117703 were selected for the breeding program against drought stress due to their higher yield and nodules in rainfed and irrigated environments. These genotypes resembled the optimal genotype more on the GGE biplot, making them ideal candidates for selection.

## Data Availability

The original contributions presented in the study are included in the article/**Supplementary Material**. Further inquiries can be directed to the corresponding author.

## Supplementary material

Supplementary Table 1 Drought tolerance score, grain yield and nodule characteristics of the moderate drought tolerant and tolerant cluster group members of chickpea germplasm. DTS, drought tolerance; GY, grain yield (g/plant); NB, nodule biomass (m³); NDW, nodule dry weight (g/plant); NFW, nodule fresh weight (g/plant).
