# Supplementary figures and images for: Selection of high nitrogen fixation chickpea genotypes under drought stress conditions using multi-environment analysis
Source: Front Plant Sci. 2025 Apr 7;16:1490080. doi: 10.3389/fpls.2025.1490080 (PMC12009914; doi:10.3389/fpls.2025.1490080)

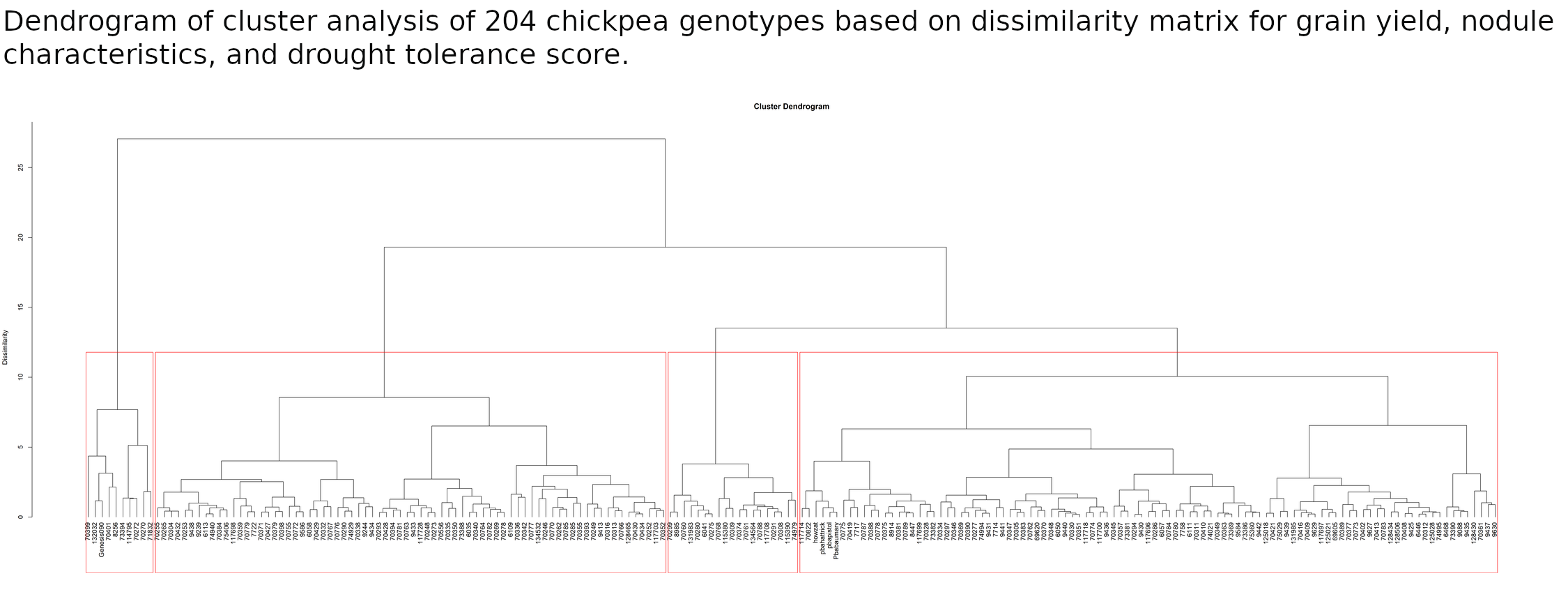

Supplement: Supplementary file 2 [file Image1.png]

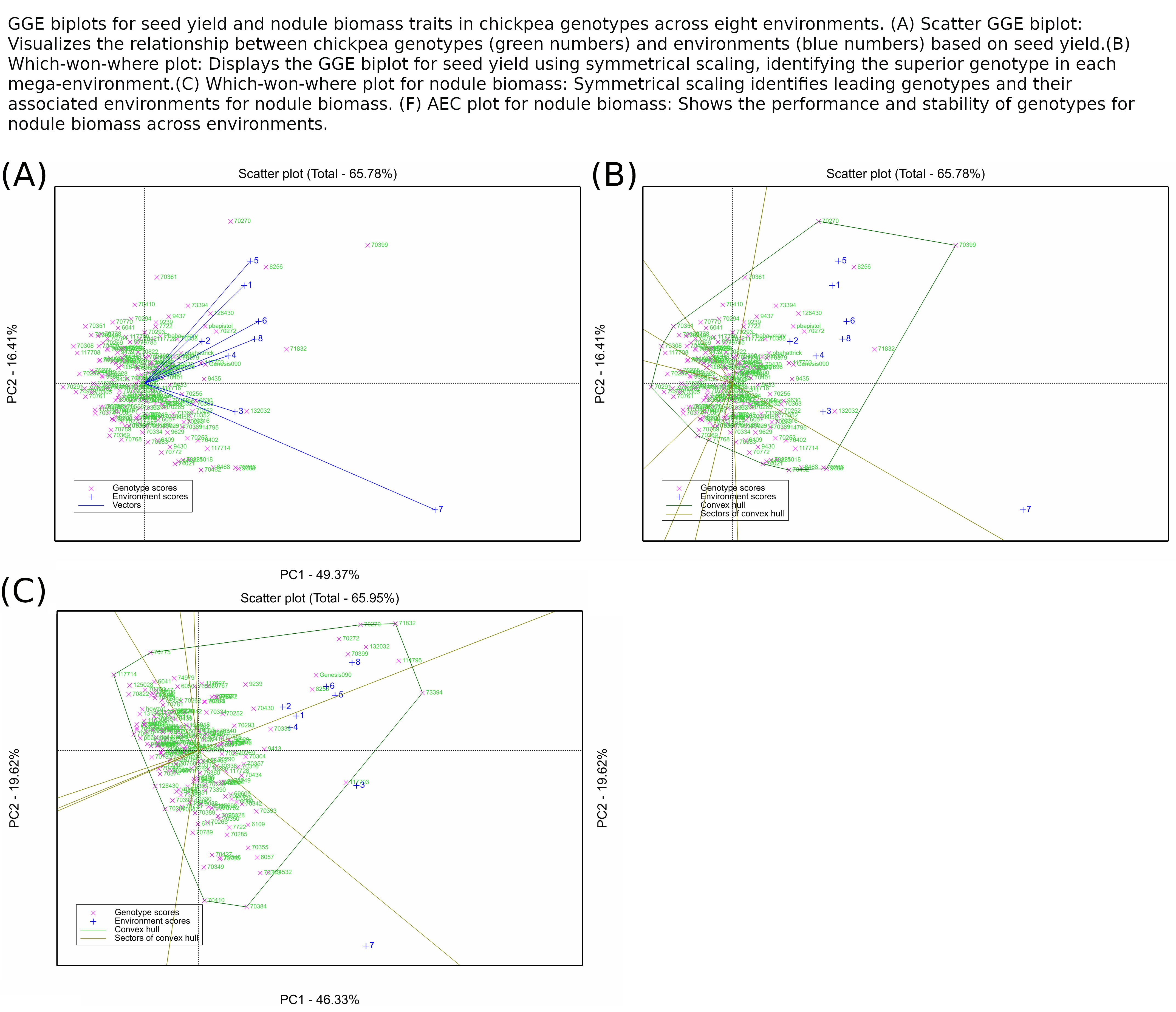

Supplement: Supplementary file 3 [file Image2.png]
